# Dynamics of neural representations when searching for exemplars and categories of human and non-human faces

**DOI:** 10.1038/s41598-018-31526-y

**Published:** 2018-09-05

**Authors:** Laurie Bayet, Benjamin Zinszer, Zoe Pruitt, Richard N. Aslin, Rachel Wu

**Affiliations:** 1 Laboratories of Cognitive Neuroscience, Boston Children’s Hospital, Boston, MA 02215 USA; 2 Department of Pediatrics, Harvard Medical School, Boston, MA 02215 USA; 3 Department of Communication Sciences and Disorders, University of Texas at Austin, Austin, TX 78712 USA; 4 Haskins Laboratories, New Haven, CT 06511 USA; 5 Department of Psychology, University of California, Riverside, CA 92521 USA

## Abstract

Face perception abilities in humans exhibit a marked expertise in distinguishing individual human faces at the expense of individual faces from other species (the other-species effect). In particular, one behavioural effect of such specialization is that human adults search for and find categories of non-human faces faster and more accurately than a specific non-human face, and vice versa for human faces. However, a recent visual search study showed that neural responses (event-related potentials, ERPs) were identical when finding either a non-human or human face. We used time-resolved multivariate pattern analysis of the EEG data from that study to investigate the dynamics of neural representations during a visual search for own-species (human) or other-species (non-human ape) faces, with greater sensitivity than traditional ERP analyses. The location of each target (i.e., right or left) could be decoded from the EEG, with similar accuracy for human and non-human faces. However, the neural patterns associated with searching for an exemplar versus a category target differed for human faces compared to non-human faces: Exemplar representations could be more reliably distinguished from category representations for human than non-human faces. These findings suggest that the other-species effect modulates the nature of representations, but preserves the attentional selection of target items based on these representations.

## Introduction

Face perception abilities in humans exhibit a marked specialization for distinguishing individual human faces, along with a relative difficulty in distinguishing faces from another species (the other-species effect^1,2^) or distinguishing between different human faces from another race or ethnicity (the other-race effect^3^). While identifying individual other-species or other-race faces tends to be difficult^2,4–7^, classifying other-species or other-race faces on the basis of their species or race tends to be quite easy^8^. The specialization of face processing abilities to the identification of own-species or own-race faces is thought to reflect the effect of early experiences in shaping high-level perceptual expertise (perceptual narrowing, for reviews, see^4,9^). At the neural level, processing of other-race faces is associated with reduced activations in the fusiform face area during encoding^10^, reduced neural repetition suppression for face identity in occipito-temporal regions around 170 ms post-onset^7^, and more negative amplitudes of the N170 (but see^11^) and N250 components^11–13^ (see also^14^).

The neural basis of attention towards other-species or other-race faces during visual search is less well established. At the cognitive level, visual search involves the representation and maintenance of the search target, a guided search for the target amongst distractors, the attentional selection of the matching stimulus, and finally the identification of the matching stimulus followed by a behavioural response^15^. At the neural level, visual search engages multiple areas spanning the parietal, frontal, and occipito-temporal cortices^16,17^. Top-down attention towards faces versus houses, presented at the same spatial location, has been associated with increased activity in the fusiform face area driven by synchrony with the inferior frontal gyrus^18^. Recent studies have revealed more negative amplitudes of the N2pc^19^ component when searching for a specific exemplar (e.g. looking for a specific digit) presented on either the left or right side of a central fixation point, compared to a broader category of objects or symbols (e.g. looking for any digit) on the left or right^20–22^, presumably reflecting the higher specificity of the search template when looking for exemplars. The same phenomenon has been described in the context of visual search for faces: looking for a specific face (e.g. looking for Bob) is associated with a larger N2pc than looking for a category of faces (e.g. looking for any male face), regardless of face species^23^. This result contrasts with the behavioural observation that category search is more effective with other-species faces than with human faces, and vice-versa for exemplar search^23^. Thus, a first question is whether a different technique, multivariate pattern analysis (MVPA) of the EEG signal, could reveal neural differences reflecting the attentional selection of exemplar and category, human and non-human face targets. That is, the attentional selection of human and non-human faces might be differentiated in these multivariate signals despite the lack of effect of species differences at the level of attentional selection as indicated by the N2pc component^24^. A second question is whether differences in the representation of human and other-species (non-human) faces might be evidenced during exemplar- and category-based visual search either preceding or following attentional selection, as predicted from studies examining the processing of human or non-human faces presented in isolation.

**Figure 1:**
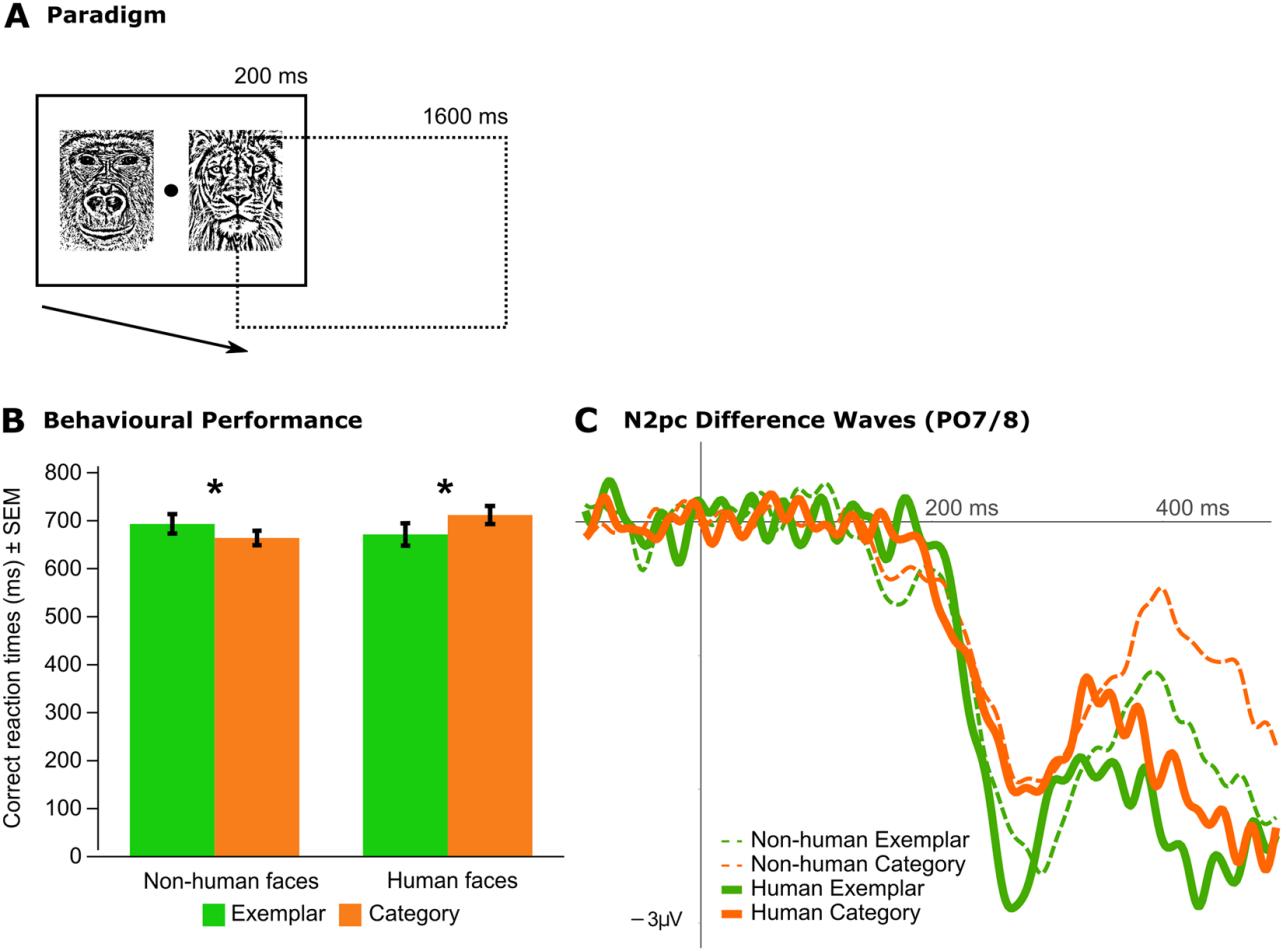
Visual search task. (**A**) Sample visual search trial. Participants searched for a specific ape (Experiment 1) or human face (Experiment 2) or for a category (any ape face in Experiment 1, or any male/female face in Experiment 2). See Wu *et al*.^23^ for specific details on the procedure. (**B**) Reaction times from the correct trials. *p < 0.05, ANOVA with planned paired t-tests. The data were previously reported in Wu *et al*.^23^. (**C**) N2pc difference waveforms obtained at PO7/8 electrodes. N2pc amplitudes were significantly larger for Exemplar than Category targets, with no effect of face species (ANOVA, see Wu *et al*.^23^).

To examine these questions, we used time-resolved MVPA with a previously collected electrophysiological dataset^23^ from 41 human adults performing a visual search task for human faces (N = 21) or non-human faces (N = 20; Table 1) to investigate the neural correlates of processing and selecting human versus non-human target faces. Time-resolved MVPA, which is more data-driven than event-related potentials (ERPs), does not rely on predefined analysis time-windows or regions of interest and can directly test representational hypotheses (i.e., information-based as opposed to activation-based)^25–28^. Thus, the MVPA approach may reveal underlying neural representations that are missed with more traditional ERP approaches. Left-right pairings of faces were presented for 200 ms followed by a 1600 ms response period during which participants indicated the target presence or absence with their right hand using arrow keys (Fig. 1A). Participants were instructed to search for either an exemplar-level target (e.g. a specific non-human or human face) or for a category-level target (e.g. any ape or female or male face). Analyses were restricted to correct trials^23^, and stimuli were identical across the category-level and exemplar-level conditions.

**Table 1:** Demographic information.

		Experiment 1: Non-human Faces	Experiment 2: Human Faces	Census data^50^ of the general local population
N		20	21	—
Gender	N Female	11	11	—
	% Female	55%	52%	52%
Age	Median age	26.5 years	21.0 years	31.4 years
	Range	19–44 years	19–31 years	—

Consistent with prior N2pc results^23^, we hypothesized that target location would be classified from the electrophysiological signal with higher accuracy for exemplar-level than category-level human target faces within the N2pc time window (200–300 ms). In addition, we aimed to test whether target location would be classified with higher accuracy for category-level vs. exemplar-level non-human target faces, as suggested by the behavioural results, or vice-versa, as suggested by the ERP results. Finally, given the greater difficulty of exemplar-level but not category-level processing of other-species and other-race faces^1–4,9^, we directly tested for the effect of task-dependent processing (i.e. exemplar-level versus category-level target processing) on neural patterns when searching for human faces versus non-human faces.

## Results

The behavioural results and event-related potential (ERP) component analyses of the present dataset have been previously reported in Wu *et al*.^23^. The results of these analyses are briefly summarized below.

### Previous behavioural and event-related potential results

Participants exhibited a canonical behavioural other-species effect, i.e. better performance for category-level non-human targets compared to exemplar-level non-human targets and vice versa for human targets^23^. Participants were faster to detect exemplar versus category human target faces, but were faster to detect category versus exemplar non-human target faces (Fig. 1B). This behavioural effect may be interpreted as resulting from perceptual expertise for human faces (perceptual narrowing^4,9^), jointly resulting in the weakening of exemplar-level processing of non-human faces and in the strengthening of exemplar-level processing of human faces^4,9,23^.

The N2pc, a posterior ERP component indexing neural target selection and contralateral to the focus of attention^15,29^, was similar when searching for and finding human and non-human faces. The N2pc had larger amplitudes for exemplar than category targets regardless of target face type (human or non-human) despite slower and more inaccurate responses to non-human exemplar targets (Fig. 1C). These results suggest that the brain retains exemplar-level sensitivity for non-human faces during attentional selection, despite behavioural differences.

How can the observer neurally select a target efficiently but still have difficulty indicating its presence or absence behaviorally? The N2pc reflects a portion of the electrophysiological signal that is related to attentional selection, but where the complete representations of the stimuli themselves are not accessible. In particular, the N2pc does not completely reflect processes either preceding or following attentional selection. Thus, one possibility is that differences between own-species and other-species face processing occur during visual search only after initial attentional selection has occurred, i.e., during a post-selection process of target identification^15^. A second possibility is that these differences affect the representations on which guided search operates, i.e., prior to attentional selection. To address these two alternatives, we used time-resolved multivariate pattern analyses (MVPA) of channel amplitudes over the entire scalp^28,30^ at each time-point in the EEG signal for own-species and other-species separately, and compared to resulting time-series of classification accuracy.

### Target selection

**Figure 2:**
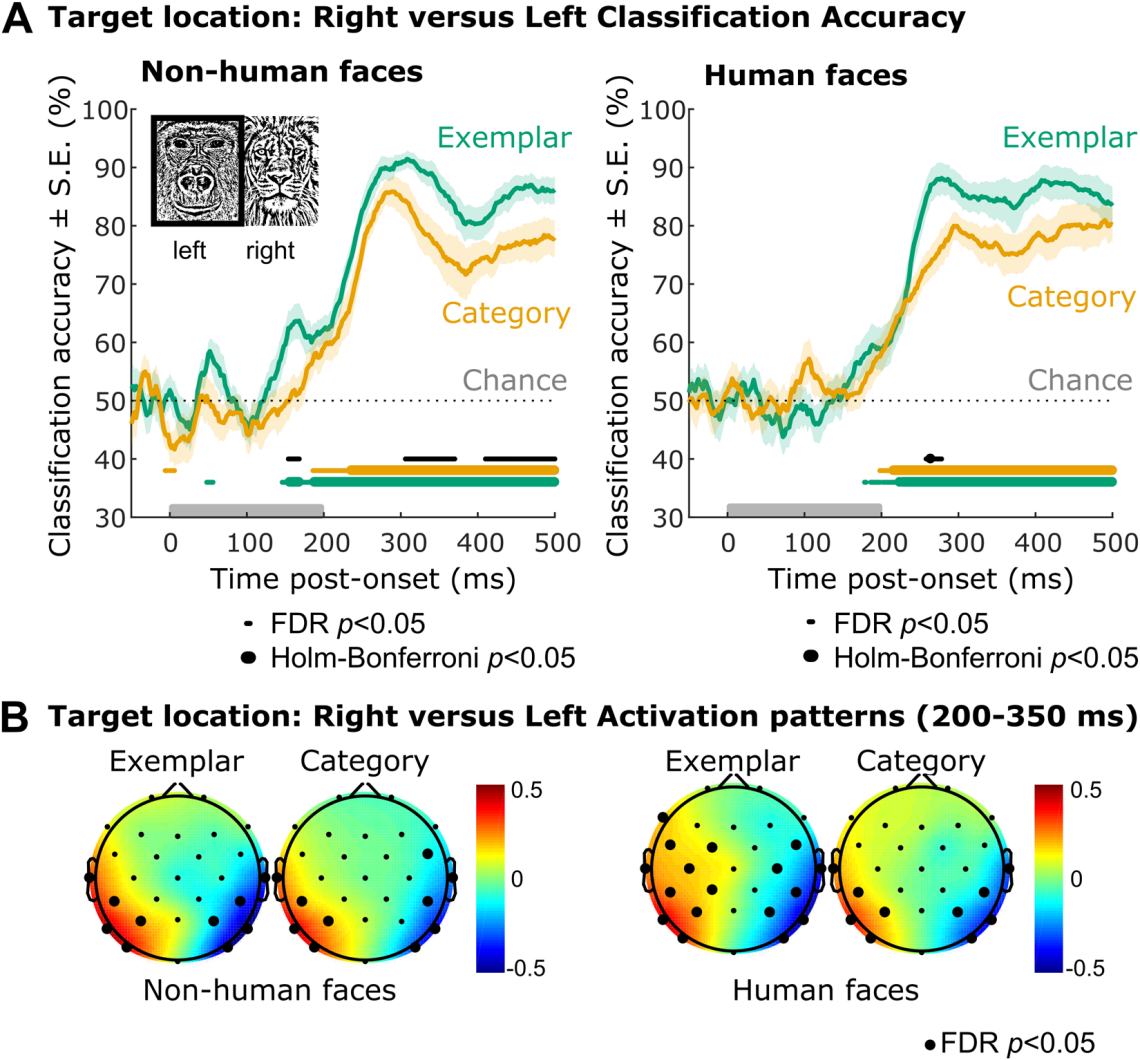
Time-resolved multivariate classification of target location. (**A**) Accuracy time-series of classifying target side (left versus right) from channel voltages within-subjects in Experiment 1 (Non-human faces, left) and Experiment 2 (Human faces, right), depending on target type (Exemplar target versus Category target). Sample-to-sample comparison of accuracies to chance (Exemplar, green; Category, orange) and across conditions (Exemplar vs. Category; black), corrected for multiple comparisons (FDR/Holm-Bonferroni, α = 0.05). See Table 2 for empirical CIs of the latencies of peak classification accuracies. (**B**) Average activation patterns^31^ supporting within-subject classification of target location from 200–350 ms post-onset, in Experiment 1 (Non-human faces, left) and Experiment 2 (Human faces, right) depending on target type (Exemplar target versus Category target). Arbitrary units. Bold circles indicate single channels with activation patterns significantly different from zero at group level after correcting for multiple comparisons (FDR, α = 0.05).

Given the canonical design of an N2pc study, we first investigated whether time-resolved multivariate pattern analyses, which necessitate fewer assumptions than ERPs, would confirm the absence of other-species effects previously observed in the N2pc results. That is, could target location be more robustly extracted from electrophysiological signals for exemplar-level than category-level search, whether participants were viewing non-human (ape) or human faces? To this end, trials were classified according to target location on the screen, either left or right, and a multivariate classifier (see Methods) was deployed at each time-point to assess whether the target location could be predicted with above-chance classification accuracy. For both human and non-human faces, classification accuracy of the target location increased from chance to peak decoding from approximately 200 ms to 300 ms, consistent with the latency of the N2pc component^15,29^. Thus, the time-course of target location classification accuracy did not differ between human and non-human faces. Classification accuracy of the target location was numerically higher for exemplar than category targets from about 200 ms post-onset for both human and non-human (Fig. 2A) faces, in line with previous N2pc results^23^. However, after correcting for multiple comparisons over all time-points, classification accuracy was only significantly higher for exemplar than category human targets at 264 ms post-onset (Fig. 2A).

Associated activation patterns were extracted^31^ (Fig. 2B), demonstrating the expected^24^ posterior left-right (lateralized) topography of the patterns that distinguished trials according to target location. Linear mixed effects models were fitted to estimate the effect of target type (exemplar versus category) and face species (human versus non-human) on peak and latency to peak target location classification accuracy. Latencies to peak classification accuracy did not reliably differ according to target type, face species, or their interaction (all *p*s > 0.1). Peak classification accuracies were significantly higher for exemplar than category targets (*t*(76) = 4.2, *p* < 0.001, 95% CI [2.18, 6.19]), with no effect of face species either alone or in interaction with target type (all *p*s > 0.1). Subgroup analyses confirmed that the effect of target type was significant for both the human face (*t*(37) = 3.34, *p* = 0.002, 95% CI [1.74, 7.09]) and non-human face (*t*(37) = 4.40, *p* < 0.001, 95% CI [2.19, 5.92]) groups separately. To provide converging evidence for the peak classification accuracies, we also analysed mean classification accuracies. Similar results were obtained when considering mean classification accuracies between 200 and 350 ms post-onset, showing higher mean accuracies during that time for exemplar than category targets with no effect of face species either alone or in interaction with target type (main effect of target type: *t*(80) = 3.51, *p* < 0.001, 95% CI [2.90, 10.51]; in the human face subgroup: *t*(40) = 2.64, *p* = 0.012, 95% CI [1.58, 11.85]; in the non-human face subgroup: *t*(38) = 3.11, *p* = 0.004, 95% CI [2.34, 11.05]). The above findings were additionally confirmed by computing non-parametric bootstrapped confidence intervals (Table 2). Thus, there was no evidence of a stronger (or faster) attentional selection of category over exemplar non-human targets at the neural level, despite faster detection at the behavioural level. On the contrary, neural responses to an exemplar target differed more reliably based on target location than did neural responses to a category target, as measured by peak classification accuracies or classification accuracies averaged over the expected latencies of target selection, regardless of whether the target belonged to a human or non-human face category. Thus, our MVPA results are completely consistent with our prior N2pc results.

**Table 2:** Empirical 95% confidence intervals.

Target location classification:	Mean peak classification latency (ms)		Mean peak classification accuracy (%)		Mean classification accuracy 200–350 ms (%)	
Non-human Exemplar	[312.60, 377.70]		[95.88, 97.97]		[79.72, 85.15]	
Non-human Category	[288.90, 362.40]		[89.30, 94.40]		[71.46, 79.92]	
*Paired difference*	[*−25.54*, *68.10*]	*NS*	[*2.95*, *7.50*]	*	[*2.55*, *10.89*]	*
Human Exemplar	[312.19, 383.91]		[93.48, 97.45]		[74.79, 83.83]	
Human Category	[294.52, 394.55]		[87.75, 94.17]		[69.15, 77.41]	
*Paired difference*	[*−41.68*, *40.54*]	*NS*	[*2.07*, *7.64*]	*	[*1.76*, *11.80*]	*
**Target type (task) classification:**	**Mean peak classification latency (ms)**		**Mean peak classification accuracy (%)**		**Mean classification accuracy 100**–**250** **ms (%)**	
Non-human	[286.09, 398.12]	*NS*	[79.58, 85.19]	*	[62.08, 69.50]	*NS*
Human	[231.03, 336.31]		[85.90, 91.89]		[68.21, 75.00]	

Lower and upper non-parametric (bootstrapped) 95% confidence intervals of the mean latency to peak classification, peak classification accuracy, and mean classification accuracy over the relevant period in Experiments 1 (Non-human face targets) and 2 (Human face targets), using 10,000 bootstrapped samples and the BCa procedure^49^. *Statistically significant difference at α = 0.05. NS Non-statistically significant difference at α = 0.05.

### Task-dependent target representation

**Figure 3:**
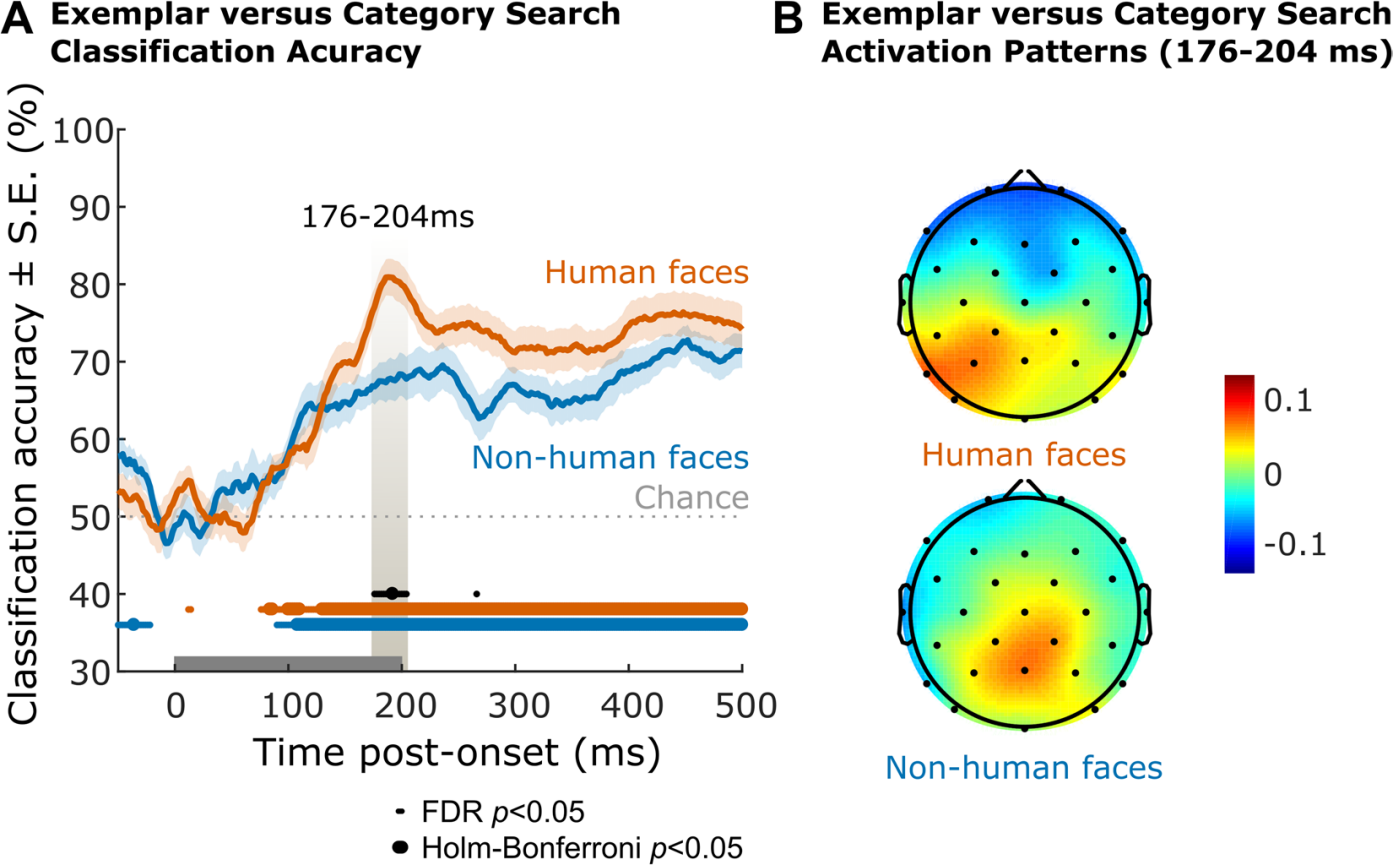
Time-resolved multivariate classification of target type. (**A**) Accuracy time-series of classifying target type (Exemplar versus Category) from channel voltages within-subjects in Experiment 1 (Non-human faces, blue) and Experiment 2 (Human faces, red), averaged over target locations. Sample-to-sample comparison of accuracies to chance (Non-human faces, blue; Human faces, red) and across conditions (Non-human vs. human faces, black), corrected for multiple comparisons (FDR/Holm-Bonferroni, α = 0.05). (**B**) Average activation patterns^31^ supporting within-subject classification of target type from 100–250 ms post-onset, in Experiment 1 (Non-human faces, bottom) and Experiment 2 (Human faces, top) averaged over target locations. Arbitrary units. No single channel had an activation pattern significantly different from zero at group level after correcting for multiple comparisons (FDR, α = 0.05).

We next derived a multivariate index of target processing at the exemplar versus category level by classifying trials according to the level at which the target search was directed, i.e. either exemplar-level or category-level. The goal was to provide an omnibus index of how different neural responses were when searching for an exemplar versus category target, which is something that could not be evaluated from the N2pc (i.e. it only assesses attentional selection). Such differences between exemplar or category searches are expected to reflect task-induced differences in target processing strategy, including the representations used for visual search. Classification accuracy for exemplar versus category target trials was significantly above chance from approximately 100 ms post onset for both human and non-human faces (Fig. 3A). Permutation tests (10,000 samples with shuffled labels) conducted at each time-point, and then corrected for multiple comparisons, demonstrated that classification accuracy was significantly higher for human than for non-human faces from 176–204 ms post-onset, i.e. at a latency preceding that of target selection (Fig. 3A; associated activation patterns Fig. 3B). Thus, neural responses associated with category versus exemplar trials differed more at 176–204 ms post-onset when participants searched for human faces than for non-human faces. Two-sided *t*-tests for independent samples on the peak classification accuracy, latency to peak classification accuracy, and mean classification accuracy at 100–250 ms post-onset confirmed this interpretation of the data. Peak classification accuracy (*t*(38.97) = 3.07, *p* = 0.004, 95% CI [2.19, 10.66]) and mean classification accuracy averaged over 100–250 ms post-onset (*t*(38.63) = 2.06, *p* = 0.046, 95% CI [0.11, 10.65]) were significantly higher in the human face group than in the non-human face group. Mean latencies to peak classification accuracy did not reliably differ between groups, although there was a non-significant trend for shorter latencies to peak classification accuracy in the human face group (*t*(38.63) −1.75, *p* = 0.088, 95% CI [−150.8606, 10.9939]). Non-parametric bootstrapped 95% confidence intervals were also run and broadly confirmed the above results, showing significantly higher mean peak classification accuracy in the human group than the non-human group, but no significant difference between the groups’ mean latency to peak classification or mean classification accuracy averaged over 100–250 ms post-onset (Table 2).

Importantly, this pattern of results persisted when strictly equating the number of valid trials available in each condition, suggesting that it cannot be attributed to differences in valid trial numbers resulting from variations in behavioural performance across task conditions (Supplementary Results and Supplementary Table S1).

## Conclusion

Multivariate pattern analyses (MVPA) of electrophysiological data during a visual search task suggest similar speed and strength of target selection for human and non-human target faces, consistent with earlier ERP analyses of a specific component (i.e. N2pc). Both of these neural findings about target *location* contrast with clear differences in target detection performance at the behavioural level favouring human faces^23^. MVPA additionally revealed that target *location* could be more accurately classified from neural patterns during exemplar search compared to category search for both human and non-human faces, even though behaviourally the participants were impaired in responding to non-human exemplar targets but not non-human category targets. Neural patterns also differed according to task, i.e. whether participants engaged in exemplar or category search. Target *type* (exemplar or category) could be classified from approximately 100 ms, with higher accuracy for human than non-human faces at a latency (approximately 175–200 ms) that preceded target selection (based on latencies to peak target location classification, Table 2).

## Discussion

The aim of the present study was to investigate the dynamics of the representations elicited during visual search, specifically for familiar (human faces) and unfamiliar (non-human faces) stimuli. First, multivariate analyses replicated the ERP results that had shown higher N2pc amplitudes (selection of target in a particular hemi-field) for exemplar than category face targets regardless of face species^23^. The present findings are also in accordance with previous reports using multivariate analyses of EEG data from a visual search task, showing that target location can be classified reliably based on the EEG signal from approximately 200 ms^24,32^. Here, we additionally show that target location can be more reliably classified from the multivariate EEG signal when the target is an exemplar than when the target is a category, regardless of target processing expertise (here, human or non-human face species). Such an effect, which extends the documented N2pc effect^23^ to a multivariate perspective, may originate from the increased specificity of exemplar targets. Second, we showed that neural responses associated with exemplar versus category level processing differed more reliably for human than non-human faces from approximately 175–200 ms after stimulus onset. A possible interpretation of this finding is that the representations of human face exemplars are more robust or engage more specialized neural processes than those of non-human face exemplars. Efficient exemplar (versus category) level processing of human but not non-human faces is a theoretical hallmark of perceptual expertise for human faces (perceptual narrowing^9^) that is most clearly evidenced behaviourally^2,5,33,34^, but also with EEG using repetition suppression^7^. The current finding provides a potential marker of perceptual expertise for human versus other-species faces, apparent at a latency (175–200 ms) that precedes target selection^35^. The results are in line with prior reports of effects at similar latencies when comparing responses to own- versus other-race faces^7,12^. Future research, using a design in which only one stimulus was present on each trial, will be needed to determine whether the effect we describe is related to the modulation of the N170 or of the later N250 component by other-race faces^11,12,14^.

Our findings also suggest that the other-species effect modulates representations preceding attentional selection during visual search, but not the strength of attentional selection that emerged from these representations. While the effect of perceptual expertise for own-species and own-race faces on perceptual discrimination is well understood, mechanisms that lead to its secondary effects on learning and cognition remain unclear. Infants, for example, are more likely to learn from own-race than other-race adults under uncertainty^36^. Here, we provide evidence that the other-species effect leaves attentional selection intact while impacting processing between 175–200 ms post-onset during visual search. Attentional selection and target identification have been proposed as two successive but independent stages in visual search, the latter relying on matching the stored representation of the target (i.e., attentional template) with that of the selected object^15^. Building on this theoretical distinction, we posit that attentional selection of other-species targets might be preserved, but their identification might be impaired as a result of difficulties in the extraction or maintenance of task-specific representations in the early phases of visual search that precede attentional selection. Although visual search theories rest on the notion that working memory representations (i.e., attentional template) guide search^15^, studies have only very recently started investigating the content of representations during visual search via multivariate analyses^24,32^. Moreover, the majority of N2pc visual search studies to date have shown a strong correlation between N2pc latency and amplitude and behavioural performance^15^. Future studies could further investigate the functional relations and dissociations between these different phases of visual search (guidance, attentional selection, and finally target identification and behavioural response), such as isolating factors that do modulate the strength of attentional selection versus target representation or identification, or what is required for an attentional template to lead to benefits in behavioural performance. It is also conceivable that the relative independence of the strength of attentional selection from perceptual expertise may originate from the fact that the attentional selection of targets in the current task was partly spatially based (i.e. left or right). Because purely object-based attention involves domain-specific activations in the extra-striate visual cortex^18^, future studies might investigate whether purely object-based (rather than partly spatially-based) attention selection is preserved or altered for other-species faces.

There were several limitations to the current study. The physical differences between the human face and non-human face stimuli are an important consideration in interpreting the results. This discrepancy is a typical concern in studies contrasting non-human and human faces (e.g.^2,37^). The stimuli differed more than usual in the current study because the non-human faces were black and white line drawings and the human faces were colour photographs. In addition, human and non-human face processing were compared in a between-subject rather than within-subject design. Despite these limitations, a canonical (behavioural) other-species effect was observed in Wu *et al*.^23^. Future studies should replicate these findings with human and non-human face stimuli that are more closely matched, preferably in a within-subject design, to confirm that the current findings generalize to other stimuli. Moreover, it is not clear how far the present findings can be attributed to face-specific processing or to the broader dimension of perceptual expertise. Similarly, it is not clear whether the primary differences we obtained were driven by category search strategies (gender/species distinction), exemplar search strategies (face identity), or both. A large body of research supports the domain-specificity of expertise effects in face perception^38^ (but see e.g.^39–42^), however N2pc studies have demonstrated that the exemplar versus category effects on attentional selection are robust across various stimulus types (e.g., letters^20^, numbers, kitchen items^22^, clothing^22^, novel alien families^43^, novel Chinese characters^21^). Future studies should determine whether the more reliable differentiation of responses to exemplar versus category tasks evidenced in the present study generalizes to other exemplar versus category contrasts relevant for face processing expertise (e.g., identity versus ethnicity) or to an increased differentiation of responses across distinct category tasks (e.g., ethnicity versus gender). Future studies that manipulate expertise within-subjects will be especially critical, such as cross-ethnicity studies of the other-race effect or training studies in which participants acquire expertise for new symbols.

Overall, the present findings suggest that the processing of other-species versus human faces differ in the specificity of perceptual representations, but not in the strength of attentional selection, in the case of visual search. We found that neural responses differed more reliably depending on task (exemplar vs. category search) for human than non-human faces. Strikingly, however, the attentional selection of left versus right targets remained strong during exemplar search even for non-human faces and despite a clear behavioural impairment. The results also demonstrate the usefulness of flexible, data-driven approaches to analysing neural data in such cases where typical electrophysiological components can be difficult to either evidence or functionally interpret, such as in the context of developmental populations or non-canonical paradigms (here, a visual search task with own- and other-species faces). The present findings contribute to demonstrating what is “lost” in the perceptual tuning of face processing to own- versus other-species faces, with implications for understanding perceptual plasticity throughout the lifespan. In addition, they provide clues about what is required for efficient top-down visual search and the functional dissociation between perceptual representations and the process of attentional selection during visual search.

## Methods

### Participants

EEG data from a total of 41 adults were included in this study (demographic information provided in Table 1). In addition, data from 17 adults were excluded due to excessive eye movement artefacts (<50% trials kept; N = 14), or poor behavioural performance (<75% accuracy; N = 3). Twenty-one participated in Experiment 1 (non-human faces), and 20 participated in Experiment 2 (human faces). All participants provided written informed consent prior to the study, all methods were carried out in accordance with the relevant guidelines and regulations, and the study was approved by the Research Subjects Review Board of the University of Rochester (Institutional Review Board).

### Face Stimuli

Stimuli for Experiment 1 were black and white line drawings of ape and other non-human animal faces created by RW (adapted from Mollison & Goodall, 2004^44^) and equated for the number of black pixels. Black and white stimuli were used to remove gross differences between ape and other non-human faces due to colour and darkness. Stimuli for Experiment 2 were a subset of those used in Rossion and Caharel (2011)^45^, which were colour photographs equated for pixel RGB intensities. Thus, stimuli characteristics were equated within each experiment, but not across the human and non-human faces experiments.

### Visual Search Task

The stimuli, design, and procedure of the visual search task have been described elsewhere^23^. Participants were presented with left/right pairings of faces on a computer screen and instructed to report the presence or absence of a target face, which could have been on the left or right of the screen. Participants used their right hand to report on the absence or presence of a target face by pressing arrow keys. Importantly, all trials included in the analysis had the same motor response (target present, correct behavioural response). Target faces were either one specific face (exemplar search) or a given category of faces (category search). Faces were presented for 200 ms, with 1600 ms ISIs. Faces were either non-human faces (Experiment 1) or human faces (Experiment 2).

In Experiment 1 (non-human faces), exemplar targets were a specific ape face, a different face for each participant, and category targets were any ape faces (versus other animal face distractors, i.e., the category task was framed as a species task). In Experiment 2 (human faces), exemplar targets were a specific male or female face, also different for each participant, and category targets were any male or female face (i.e., the category task was framed as a gender task), with the gender of the target faces being counterbalanced across participants.

Trials were presented in blocks of 62 exemplar search trials (including 28 target trials, half of which were presented on the left and the other half on the right side of the screen) or 56 category search trials (including 28 target trials, half of which were presented on the left and the other half on the right side of the screen), with 11 blocks of each type leading to a total of 1298 trials. Exemplar and category search trials were blocked to avoid low accuracy rates in an already difficult task^21^. Task order was counterbalanced across all participants. Participants were informed of the search type (exemplar or category search) at the beginning of each block. All non-target or incorrect target trials were rejected from later analysis.

### Software

Data pre-processing and analyses were performed in MATLAB 2013b and EEGLAB 13.5.4^46^.

### EEG Data Recording and Pre-processing

The EEG data were collected during the task at 500 Hz with a 32-ch Brain Products actiCHamp system^23^ with the left earlobe as the online reference. Robust average reference, line noise removal, and bad channel interpolation were performed using the PREP pipeline^47^. The EEG data were filtered at 0.2–200 Hz, smoothed with a 20 ms running average, and epoched from −100 to +500 ms relative to the onset of the faces^23^. Epochs were rejected for artefact if any horizontal EOG exceeded ±25 μV, any vertical EOG exceeded ±60 μV, or any other channels exceeded ±80 μV^23^. Raw voltage values at each channel and trial were baseline corrected and z-scored in reference to the baseline period. Importantly, individual trials were not averaged prior to MVPA analysis (i.e., the MVPA analysis started from single trials^28^).

### EEG Data Analysis

Electrodes used for the previously published N2pc analysis were PO7/8^23^. All scalp channels (inclusive of PO7 and PO8) were included for MVPA. The pre-processed EEG data were classified for each subject (within-subject classification) at each time-point post-onset independently (time-resolved MVPA^28^) using linear support vector machines (SVMs) implemented with libsvm-3.11^48^ in MATLAB 2013b, with 4-fold cross-validation (i.e., separation of the data into 4 folds, 3 of which were used to train the SVMs while the remainder was used to derive classification accuracies), pseudo-averaging (i.e., trials from each fold were averaged together to increase signal-to-noise ratio), and 100 permutations (i.e., random assignments of the data into each fold)^30^. The average number of valid (correct and artefact-free) trials per class (e.g. exemplar search, left side target) for each participant and classification was 111.65, corresponding to an average of 83.41% of correct trials (the remaining of which were excluded for artefacts). Statistical testing of time-resolved group comparisons of classification accuracies (Task-dependent target representation; Fig. 3A) were performed using a permutation test based on the empirical distribution of the *t*-statistic of the effect of group under the null hypothesis (*t*-test for independent samples with unequal variance, 10,000 samples with permuted group labels, one-sided empirical *p*-value). Other statistical tests were performed using paired *t*-tests, independent *t*-tests, linear mixed-effect models (see below), and/or non-parametric bootstrapped 95% CIs (10,000 bootstrap samples, BCa procedure^49^) as appropriate. In particular, comparisons of classification accuracy time-series against chance were performed using *t*-tests at each time-point, correcting for multiple comparisons across time-points^28^. P-values were corrected for multiple comparisons using the False-Discovery Rate and/or the Holm-Bonferroni method, as appropriate.

### Linear Mixed Effect Models

Linear Mixed Effects (LME) Models were fitted in Matlab 2013b, with a random intercept for each participant. Random slopes for within-subject factors (trial type) were dropped based on likelihood ratio tests on nested models. Outliers were identified and rejected based on visual inspection of the residuals distribution from the full model with no random effects (latency to peak classification accuracy: 2.44% data-points excluded; peak classification accuracy: 4.88% data-points; mean classification accuracy 200–350 ms: 0% data-points). Fixed effects were selected based on likelihood ratio tests on nested models. Model assumptions, including normality, were confirmed from visual inspection of the residuals from the best fitted model for each outcome variable.

## Electronic supplementary material

Supplementary Results

## Data Availability

The datasets analysed during the current study are available online in the following Figshare repository: 10.6084/m9.figshare.5119966.
