# Supplementary material for: Dynamics of neural representations when searching for exemplars and categories of human and non-human faces
Source: Sci Rep. 2018 Sep 5;8:13277. doi: 10.1038/s41598-018-31526-y (PMC6125483; doi:10.1038/s41598-018-31526-y)
Supplement: Supplementary file 1 — Supplementary Results [file 41598_2018_31526_MOESM1_ESM.docx]

Dynamics of neural representations when searching for exemplars and categories of human and non-human faces.

Laurie Bayet, Benjamin Zinszer, Zoe Pruitt, Richard N. Aslin, & Rachel Wu.

**Supplementary Results**

Classification analyses were restricted to correct trials only, while there were differences in behavioural accuracy between conditions. Because the classification involved pseudo-averaging of trials into a set number of 4 pseudo-trials in each class (4-fold cross-validation) at each cross-validation permutation, the *theoretical* chance level for classification was always 50% regardless of the number of available, correct trials in each class. However, this situation could still have affected *empirical* chance levels or otherwise driven classification results (as more data is likely to result in higher classification accuracy).

To formally test whether differences in trial numbers were driving the differences between groups, we have run the same classification analyses, keeping the number of trials the same for the classified conditions, within and across participants, discarding data from participants with less than a set number of trials in each condition and randomly discarding excess trials from participants who had more.

Because doing so leads to a trade-off between the set number of trials available to train the classifiers and the number of retained participants in both groups, we ran these analyses with 3 different fixed numbers of trials per condition (60, 70, or 80); these represent about 40% or more of the maximum possible number of trials per condition (154), and led to keeping N=16 or more participants in each group and condition.

For each analysis, we attempted to replicate the core effects reported in the main paper, i.e. (a) higher classification accuracy for classifying exemplar than category target location (left or right), regardless of whether these targets are human or non-human faces; (b) higher classification accuracy for classifying the task (exemplar or category search) in the human than non-human faces group.

All 95% confidence intervals (CIs) were obtained empirically from 10,000 bootstrap samples. Differences between classification accuracy for classifying task (exemplar versus category search) with human and non-human faces are evaluated using *t*-tests for independent samples with unequal variance. The main effect of condition (exemplar or category search) on the classification accuracy for classifying target location (left versus right) is evaluated in a linear mixed effects model also including a main effect of group (human or non-human) and a random intercept for participant. Extreme values were excluded for linear mixed model fits based on the distribution of residuals (0-4.88% data-points). All *p*-values are corrected for multiple comparisons at the FDR level over the 12 tests.

The results of these supplementary analyses are presented in Supplementary Table S1 below. Overall, these analyses suggest that the core results of the main paper probably cannot be attributed to differences in trial numbers between conditions or groups, as they persist numerically in all cases, and statistically in at least one out of three cases where these numbers are strictly equated at the expense reducing the number of trials and/or participants, after controlling for multiple comparisons.

**Supplementary Table S1.** Replication of the main findings of the paper using strictly balanced datasets with **A.** 60 trials per condition **B.** 70 trials per condition and **C.** 80 trials per condition.

| **A. 60 trials per condition** | | | | | | | | | |
| --- | --- | --- | --- | --- | --- | --- | --- | --- | --- |
| **Target Location** | | | **Non-human Exemplar** | **Non-human Category** | | **Human Exemplar** | **Human Category** | **Main effect of Condition** | |
| N | | | 17 | 20 | | 20 | 20 |  | |
| Peak accuracy | M | | 89.24 | 84.33 | | 88.75 | 83.68 | *F*(1,71)= 9.81 *p* = 0.006 ** | |
|  | CI | | [86.00 92.24] | [80.85 87.30] | | [84.04 92.78] | [80.73 87.10] |  |  |
| Mean accuracy 200-350ms | M | | 72.39 | 64.22 | | 71.11 | 64.51 | *F*(1,74)= 11.03 *p* = 0.006 ** | |
|  | CI | | [69.22 75.69] | [59.76 68.48] | | [65.35 76.55] | [60.61 69.16] |  |  |
| Interpretation | | | Effect is numerically replicated, and statistically significant. | | | | | | |
| **Task** | | | **Non-human** | | | **Human** | | **Difference** | |
| N | | | 17 | | | 20 | |  | |
| Peak accuracy | M | | 76.24% | | | 78.91% | | *t*(30.28)=0.97  *p* = 0.371 NS | |
|  | CI | | [73.49 78.82] | | | [74.29 83.30] | |  |  |
| Mean accuracy 100-250ms | M | | 57.54% | | | 60.57% | | *t*(31.46)=1.23  *p* = 0.273 NS | |
|  | CI | | [54.79 59.88] | | | [56.55 64.53] | |  |  |
| Interpretation | | | Effect is numerically replicated, but not statistically significant. | | | | | | |
| **B. 70 trials per condition** | | | | | | | | |  |
| **Target Location** | | | **Non-human Exemplar** | **Non-human Category** | | **Human Exemplar** | **Human Category** | **Main effect of Condition** |  |
| N | | | 17 | 20 | | 19 | 20 |  |  |
| Peak accuracy | | M | 89.29 | 84.00 | | 89.26 | 86.48 | *F*(1,69)= 14.04 *p* = 0.002 ** |  |
|  |  | CI | [86.32 91.88] | [80.53 87.23] | | [84.42 92.66] | [82.50 89.95] |  |  |
| Mean accuracy  200-350ms | | M | 71.89 | 66.44 | | 70.00 | 65.20 | *F*(1,73)= 5.37 *p* = 0.035 * |  |
|  |  | CI | [67.88 74.89] | [61.16 71.07] | | [64.54 75.42] | [61.49 70.16] |  |  |
| Interpretation | | | Effect is numerically replicated, and statistically significant. | | | | | |  |
| **Task** | | | **Non-human** | | **Human** | | | **Difference** | |
| N | | | 17 | | 19 | | |  | |
| Peak accuracy | M | | 77.63% | | 82.72% | | | *t*(33.57)=2.56  *p* = 0.026 * | |
|  | CI | | [75.52 80.42] | | [79.97 85.87] | | |  |  |
| Mean accuracy 100-250ms | M | | 59.12% | | 64.89% | | | *t*(33.94)=2.91  *p* = 0.013 * | |
|  | CI | | [55.91 61.36] | | [62.47 67.92] | | |  |  |
| Interpretation | | | Effect is numerically replicated, and statistically significant. | | | | | | |
| **C. 80 trials per condition** | | | | | | | | | |
| **Target Location classification** | | | **Non-human Exemplar** | **Non-human Category** | | **Human Exemplar** | **Human Category** | **Main effect of Condition** | |
| N | | | 16 | 20 | | 19 | 18 |  | |
| Peak accuracy | M | | 92.72 | 85.23 | | 91.29 | 87.28 | *F*(1,67)=18.64 *p* = 0.006 ** | |
|  | CI | | [89.84 95.09] | [81.80 88.05] | | [86.00 94.42] | [82.97 91.03] |  |  |
| Mean accuracy  200-350ms | M | | 75.06 | 69.25 | | 70.56 | 64.64 | *F*(1,68)= 10.40 *p* = 0.006 ** | |
|  | CI | | [70.75 78.58] | [65.02 73.23] | | [64.20 76.24] | [60.00 70.34] |  |  |
| Interpretation | | | Effect is numerically replicated, and statistically significant. | | | | | | |
| **Task classification** | | | **Non-human** | | | **Human** | | **Difference** | |
| N | | | 16 | | | 17 | |  | |
| Peak accuracy | M | | 79.86% | | | 83.99% | | *t*(29.80)=1.82  *p* = 0.105 NS | |
|  | CI | | [77.25 82.66] | | | [80.71 87.37] | |  |  |
| Mean accuracy 100-250ms | M | | 62.04% | | | 64.46% | | *t*(30.61)=0.85  *p* = 0.403 NS | |
|  | CI | | [58.65 65.81] | | | [60.11 68.27] | |  |  |
| Interpretation | | | Effect is numerically replicated, but not statistically significant. | | | | | | |
